# A novel paradigm for identifying eye-tracking metrics associated with cognitive control during driving through MEG neuroimaging^☆^

**DOI:** 10.1016/j.trf.2025.103434

**Published:** 2025-11-08

**Authors:** Thomas Seacrist, Elizabeth A. Walshe, Shukai Cheng, Emily Brown, Charlotte Birnbaum, Victoria Kaufman, Flaura K. Winston, William C. Gaetz

**Affiliations:** a Department of Bioengineering, University of Pennsylvania, Philadelphia, PA, USA; b Center for Injury Research and Prevention, Children’s Hospital of Philadelphia, Philadelphia, PA, USA; c Department of Pediatrics, Perelman School of Medicine, University of Pennsylvania, Philadelphia, PA, USA; d Lurie Family Foundations’ MEG Imaging Center, Department of Radiology, Children’s Hospital of Philadelphia, Philadelphia, PA, USA; e Department of Radiology, Perelman School of Medicine, University of Pennsylvania, Philadelphia, PA, USA

**Keywords:** Eye-tracking, Neuroimaging, MEG, Simulated driving, Young drivers, Cognitive control

## Abstract

Understanding the neurocognitive underpinnings of driving behavior in adolescents is critical to improving road safety. To address this, we established a novel paradigm linking magnetoencephalography (MEG)-recorded frequency-specific brain activity to simulated driving performance, identifying periods of increased cognitive control. However, this initial paradigm did not incorporate eye-tracking – a potentially scalable proxy for cognitive control that could be leveraged by in-vehicle driver monitoring systems. This proof-of-concept study expands our paradigm by integrating eye-tracking to identify scanning behavior metrics associated with periods of increased cognitive control validated by MEG. Typically developing adolescents *(n* = 11; mean age = 15.1 ± 1.5 yrs) completed three driving tasks of varying cognitive demand, and MEG frequency specific analysis confirmed periods of high (Hi) and low (Lo) cognitive control via the established biomarker of frontal midline theta (FMT). Fixation count, fixation duration, horizontal/vertical mean gaze position, saccade amplitude, and horizontal/vertical spread of search were compared between Hi vs. Lo periods of cognitive control. Task-specific differences in fixation count (p < 0.05), mean gaze position (p < 0.01), saccade amplitude (p < 0.05), and spread of search (p < 0.01) were observed between Hi compared to Lo cognitive control periods. These differences corresponded to expected task-specific changes in scanning behavior that would accompany cognitive control over behavior, suggesting a signal that eye-tracking may serve as a proxy for underlying neurocognitive processes. This integrated approach demonstrates methodological rigor and offers a promising framework for further research and informing development of in-vehicle driver monitoring systems for detecting cognitive deficits in real time, with implications for enhancing teen driver safety.

## 1. Introduction

Motor vehicle crashes among young drivers are largely attributed to driver error, rather than reckless behavior (McKnight & McKnight, 2003). Driver error accounts for an estimated 76–96 % of motor vehicle crashes and young new drivers commit more safety–critical driving errors than more experienced drivers (Curry et al., 2011; Seacrist et al., 2021). Recognition errors – including inadequate surveillance and inattention – have been suggested as the leading cause of crashes among young drivers (Curry et al., 2011; Lestina & Miller, 1994; McKnight & McKnight, 2003; Seacrist et al., 2021). While driving experience is a contributing factor, these errors may also be attributed to frontal-lobe cognitive abilities (Ross et al., 2015; Walshe et al., 2017), some of which are still maturing in adolescence and into adulthood (Satterthwaite et al., 2013).

These recognition and inattention errors are considered errors of situational awareness, or the ability to comprehend the surrounding environment and anticipate future events (Endsley, 1988), which is critical for safe driving. The three-layer definition of situational awareness – perception, comprehension, and projection – captures how situational awareness depends on working memory, attention allocation, and cognitive processes that integrate sensory inputs with stored knowledge and goals (Endsley, 1995). Poor situational awareness stemming from errors in cognitive processing can lead to riskier driving, such as the aforementioned inadequate surveillance and inattention errors observed among young drivers. Understanding the cognitive control mechanism underlying driving in adolescence is particularly important, where ongoing neurodevelopment and limited driving experience compound the risk to safety. Detecting in-the-moment lapses in cognitive processing is also critical for enhancing in-vehicle driver monitoring systems and intervening to improve driver safety (EuroNCAP, 2022).

Recent developments in eye-tracking and neuroimaging are paving the way for new approaches to understanding cognitive control during driving. Eye-tracking is an objective method that can be used by in-vehicle driving monitoring systems to infer levels of situational awareness and cognitive processing by indicating whether drivers are scanning critical areas of the driving scene (e.g. mirrors, potential hidden hazards). Prior studies have demonstrated the relationship between eye-tracking and situational awareness in a variety of real-world tasks (Argyle et al., 2020; Hasanzadeh et al., 2016; Moore & Gugerty, 2010; Van De Merwe et al., 2012; Yu et al., 2014). For driving specifically, eye-tracking has been linked to both situational awareness (Liang et al., 2021) and cognitive processing (Harbluk et al., 2007; Recarte & Nunes, 2003). In addition, systematic reviews of hazard perception – the ability to detect or identify hazards – show that scanning behavior indexes situational awareness, which varies with age and experience (Cao et al., 2022). Together, these findings support the use of eye-tracking as a proxy for detecting the state of cognitive processes underlying situational awareness in driving. However, this needs to be validated with concurrent neuroimaging to confirm the cognitive control mechanisms.

In neuroimaging studies, fNIRS has demonstrated increased prefrontal cortex activity with increased driving complexity and cognitive workload (via secondary tasks), and associated changes in horizontal spread of search (Foy et al., 2016; Foy & Chapman, 2018). Other studies using fMRI also reveal changes in eye behavior during increased cognitive workload via distracted task paradigms during driving (Yuen et al., 2021). However, both fNIRS and fMRI approaches lack temporal resolution for capturing the rapid, event-specific changes in prefrontal cortex activity required for real-time assessment. On the other hand, EEG imaging provides an opportunity for higher temporal resolution of cognitive responses to the driving task, but studies to date largely also focus on understanding mental workload through distraction and multi-task manipulation paradigms to reveal effects (Aygun et al., 2022; Das Chakladar & Roy, 2024; Liu et al., 2023; Wascher et al., 2023; Yuen et al., 2021). Despite magnetoencephalography (MEG)’s strengths in spectral, temporal, and spatial resolution, its use in driving-related cognitive neuroscience has been limited and methodologically constrained. Prior studies either lacked frequency-specific analysis of cognitive control signals (Fort et al., 2010) or collapsed neural activity over extended driving periods, obscuring temporal dynamics (Sakihara et al., 2014). These gaps highlight the need for paradigms that integrate MEG with ecologically valid simulated driving and eye-tracking to resolve frontal lobe cognitive control in real time while driving.

To address this challenge, we recently established a novel paradigm for identifying increased cognitive control during driving by linking MEG-recorded frequency-specific brain activity to simulated driving performance (Walshe et al., 2019; Walshe et al., 2023). Specifically, we demonstrated increased frontal midline theta (FMT) activity – an established marker of cognitive control over behavior (Callaghan et al., 2017) – when braking in response to a traffic light (requiring top-down cognitive control) relative to rest. However, like other prior studies, this study did not include eye-tracking recording. Thus, in this proof-of-concept study, we incorporate eye-tracking into our existing paradigm and aim to demonstrate the utility of this new paradigm to identify eye-tracking metrics associated with periods of increased cognitive control over behavior during simulated driving. Building on our prior study, we hypothesize that eye-tracking will vary between periods of high and low cognitive control over behavior. Eye-tracking metrics may proxy the previously observed FMT higher cognitive control activity during braking – which may be detectable via in-vehicle driving monitoring systems. If we can link fundamental neurocognitive capacities (e.g., frontal lobe cognition important for situational awareness) with driver behaviors that are detectable via in-vehicle eye-tracking and vehicle telematics, we may be able to (1) detect deficits in teen driver cognitive function and (2) augment teen driver situational awareness, helping to reduce the number of crashes due to these errors.

## 2. Method

This study was approved by the Institutional Review Board at the Children’s Hospital of Philadelphia.

### 2.1. Population

Participant recruitment occurred from August 2021 through August 2023. Eleven typically developing adolescents (*Mean Age*: 15.1 ± 1.5 yrs; *Sex*: 4 Female, 7 Male) were scanned. Parental consent and participant assent were obtained. Exclusion criteria were previous diagnosis of autism spectrum disorder, Asperger’s syndrome, pervasive developmental delay, other psychiatric disorders, seizure and neurologic disorders, severe claustrophobia, or uncorrectable hearing or vision issues. After consent procedures, participants were acclimated to the experimental set-up.

### 2.2. Experimental procedures

This study utilized a novel, established, paradigm of an event-based simulated drive, operated via MEG compatible driving simulator hardware, and now paired with eye-tracking recording to characterize the eye-tracking correlates of cognitively demanding driving tasks. After consent procedures, participants were positioned in the MEG and eye tracking calibration procedures were conducted, before participants were acclimated to the MEG-compatible driving simulator hardware (Current Designs, Inc., USA) and the simulation (Carnetsoft BV, The Netherlands) via a practice trial for the driving task (described below). Once participants were familiarized with the vehicle controls, participants then completed the experimental driving task. In summary, participants performed the same prototypical simulated driving task repeated over numerous trials, for an event-related approach to examining brain behavior: containing embedded and time-stamped events corresponding to traffic light changes, wind gusts, and lead vehicle braking as well as driver behavior (e.g., acceleration, braking, and steering). Following the experimental driving task, participants completed a magnetic resonance imaging brain scan for anatomic localization of MEG-detected brain activity.

### 2.3. MEG data Acquisition and preprocessing

Participants underwent MEG scanning while seated upright and using the MEG compatible driving simulator. Whole-head MEG recordings were acquired using a 275-channel CTF Omega system (CTF MEG International Services, Coquitlam, BC), sampled continuously at 600 Hz with a 0–150 Hz band-pass filter. Participants sat upright in the magnetically shielded room. Three localization coils were affixed at the nasion and pre-auricular points for continuous head-position tracking (10 Hz) and for co-registration with each participant’s anatomical MRI.

MEG data were epoched relative to trial onset and driving events of interest. Third-order synthetic gradient correction was applied, and trials exceeding 1 cm head motion were excluded. The MRI brain scan provided anatomic localization of MEG-detected brain activity. Preprocessing steps (including artifact rejection and DC offset removal) followed the procedures detailed in Walshe et al., 2023, which also provides a full description of our analytic pipeline.

### 2.4. Meg-compatible driving simulator hardware

The hardware for driving simulation consisted of a MEG-compatible projection screen paired with a MEG-compatible driving hardware including a steering wheel, brake, and accelerator pedals (for more detailed description see Walshe et al., 2023). The MEG compatible driving hardware interfaces with the Carnetsoft BV simulation software which delivers the driving scenarios for this study, described in detail below. Participants viewed the simulated driving tasks on a screen ~ 90 cm in front of the seat, using a back-projected display (via mirrors), controlled via a simulation presentation computer outside the magnetically shielded room.

**Fig. 1.**
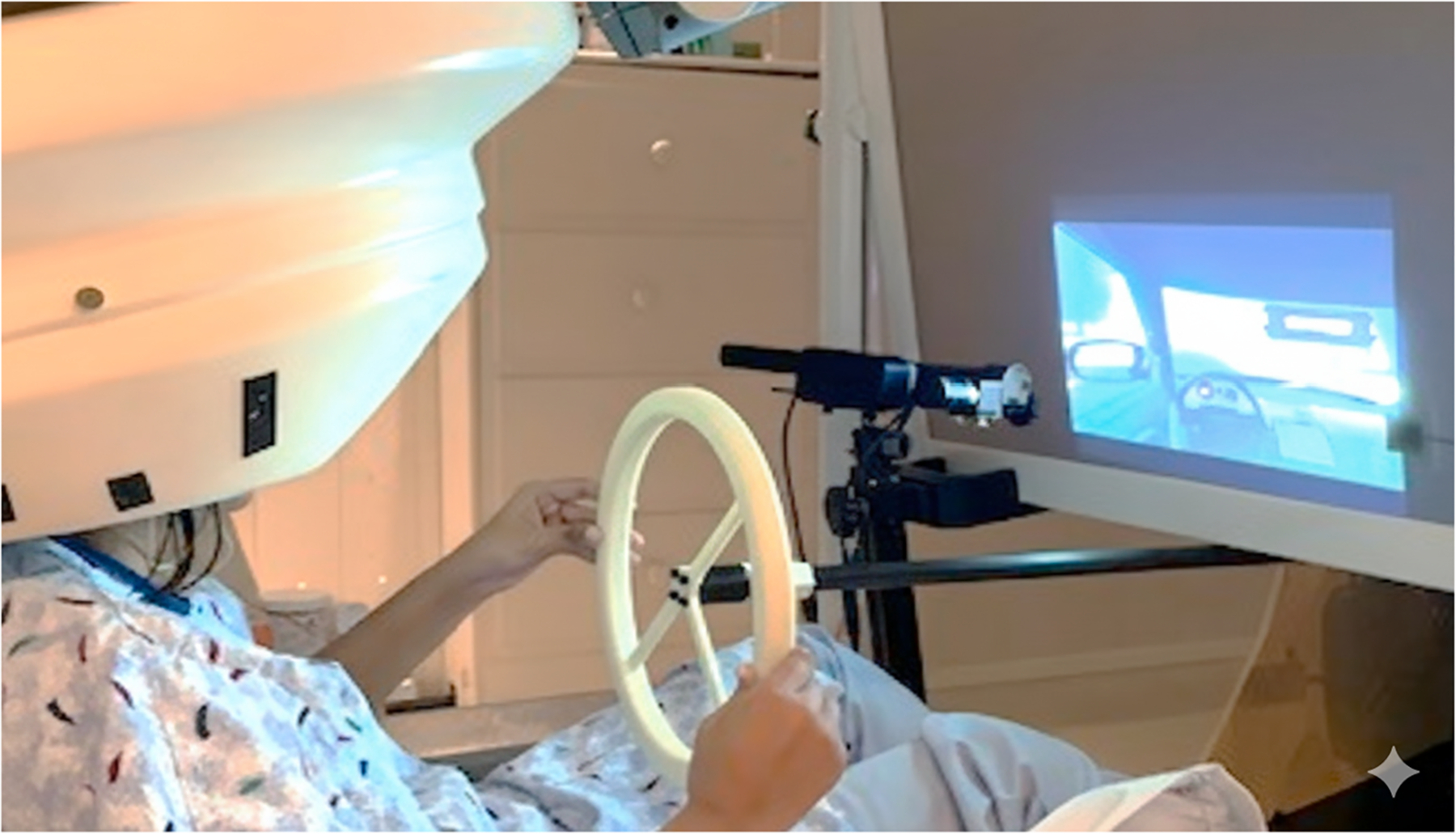
Exemplar participant using the driving simulation in the MEG laboratory with a MEG-compatible eye-tracker.

**Fig. 2.**
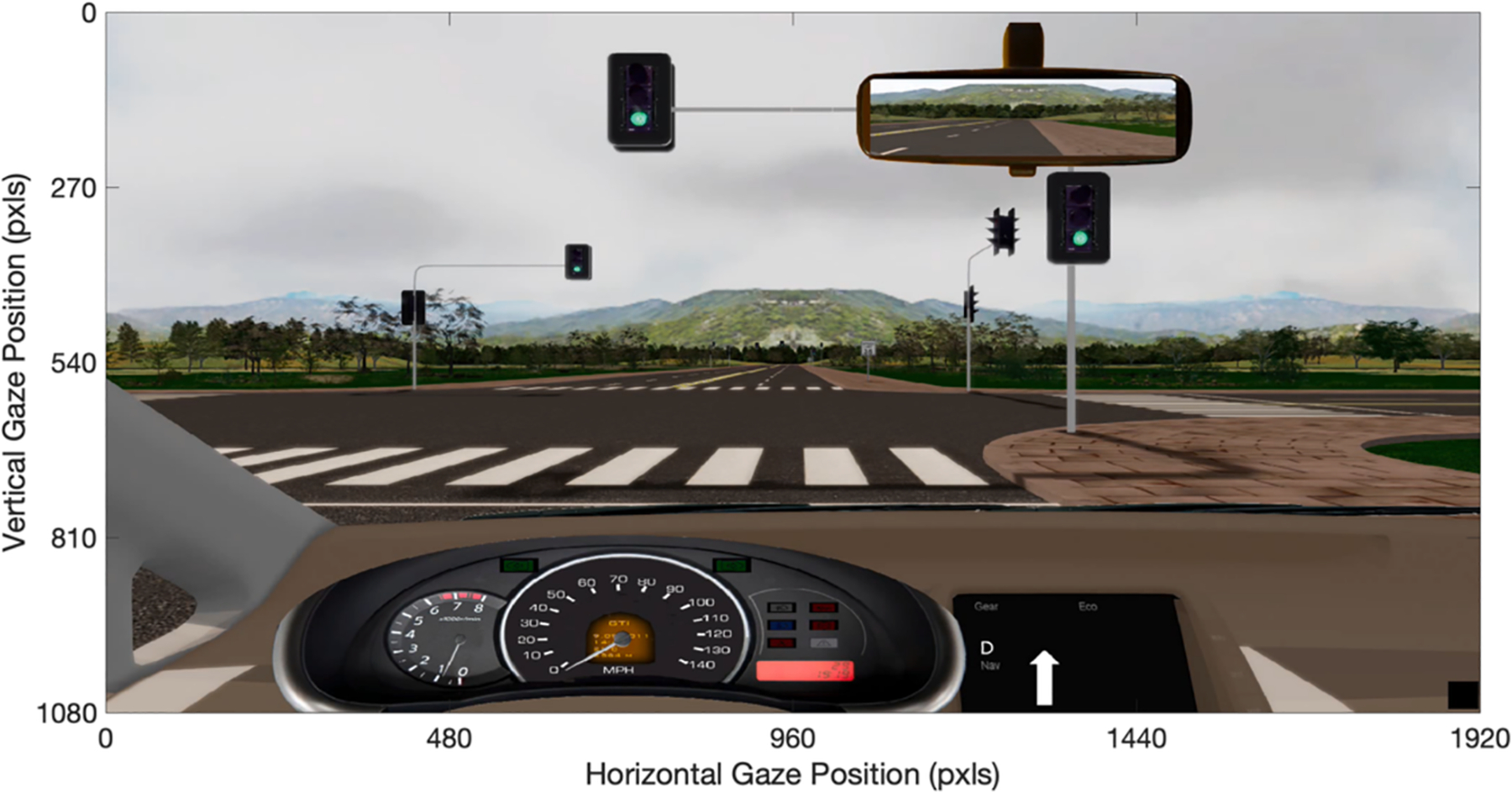
Exemplar simulated driving scene with pixel origin located in the upper right corner of the screen.

On-screen gaze position was collected at 1000 Hz using a MEG-compatible SR Research EyeLink 1000 (SR Research, Canada) that was fixed to the bottom of the projection screen (see Fig. 1). The SR Research EyeLink 1000 includes a Fiber Optic Camera Head, which allows the system to track eye movement in sensitive environments such as MRI and MEG installations. Fixations and saccades were identified by the EyeLink software using default thresholds: saccades were defined as any eye motion exceeding 30°/s in velocity or 8000°/s^2^ in acceleration whereas fixations were defined as eye motion below both thresholds. Gaze points were output in pixels, with the upper left corner of the screen being defined as the origin (0,0), shown in Fig. 2.

### 2.5. Driving simulation

Participants drove three repeated-trial prototypical driving scenarios, each requiring relatively varying levels of required cognitive workload, in the following sequential order: (1) Unanticipated Steering task (moderate workload), (2) Traffic Light Braking task (low workload), and (3) a Lead Car Braking task (high workload). Seven participants completed all three driving tasks.

#### 2.5.1. Unanticipated Steering task

**Fig. 3.**
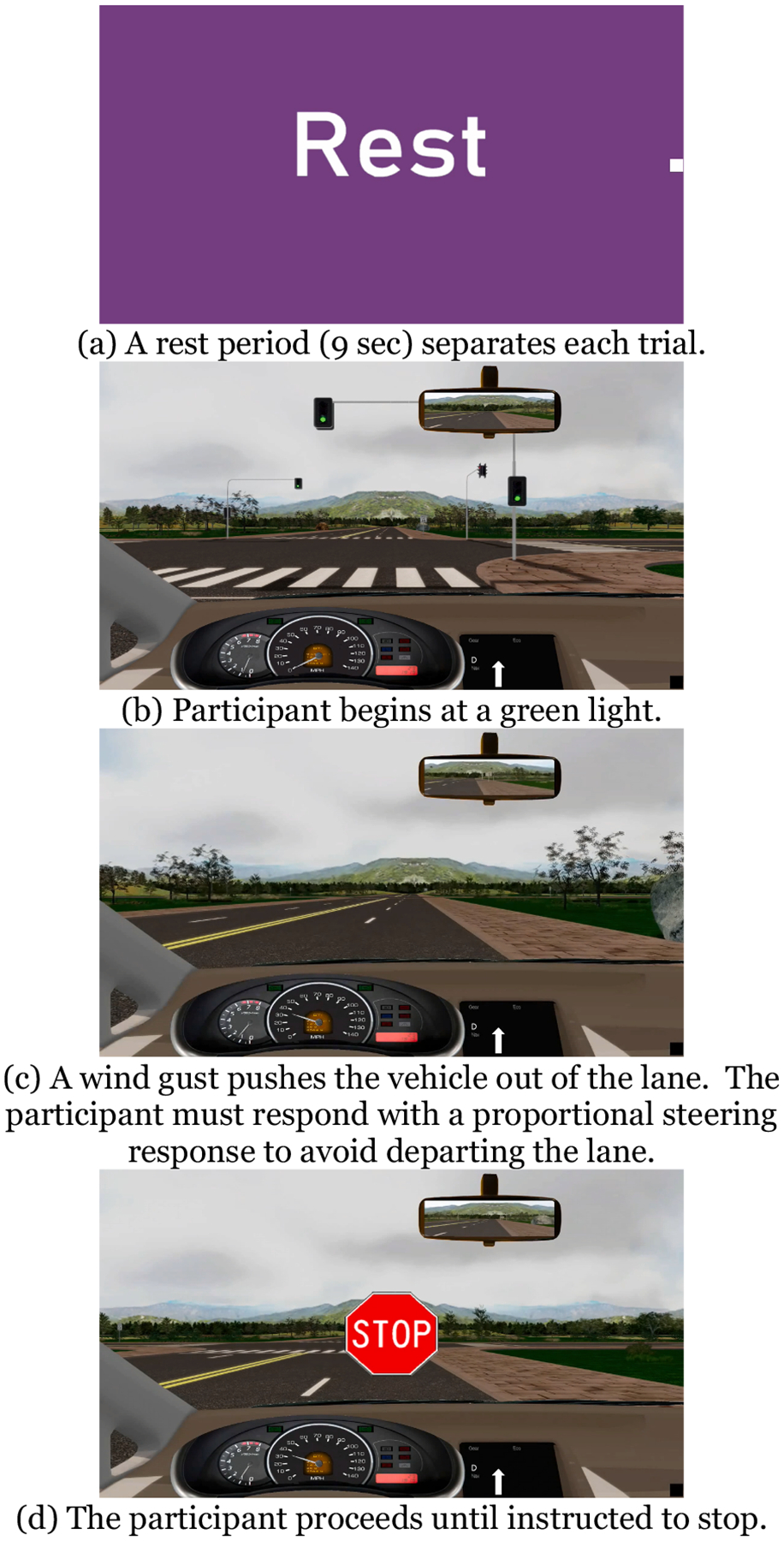
Unanticipated Steering Task.

The Unanticipated Steering task is depicted in Fig. 3. During this moderate cognitive workload task, the participant begins at a green light, stopped. The participant drives straight in his lane until instructed to stop by a visual that appears on the screen (~20 s of driving). During the drive, a wind gust of varying direction and intensity with no warning pushes the vehicle out of its lane. The participant must conduct a proportional steering response to maintain lane position. No distractions or ambient traffic are present. The task is repeated for 20 trials, with a 9 sec rest period between each trial. Participants were provided two practice trials with no wind gust and were instructed to drive until told to stop while maintaining speed and lane position.

#### 2.5.2. Traffic Light Braking task

**Fig. 4.**
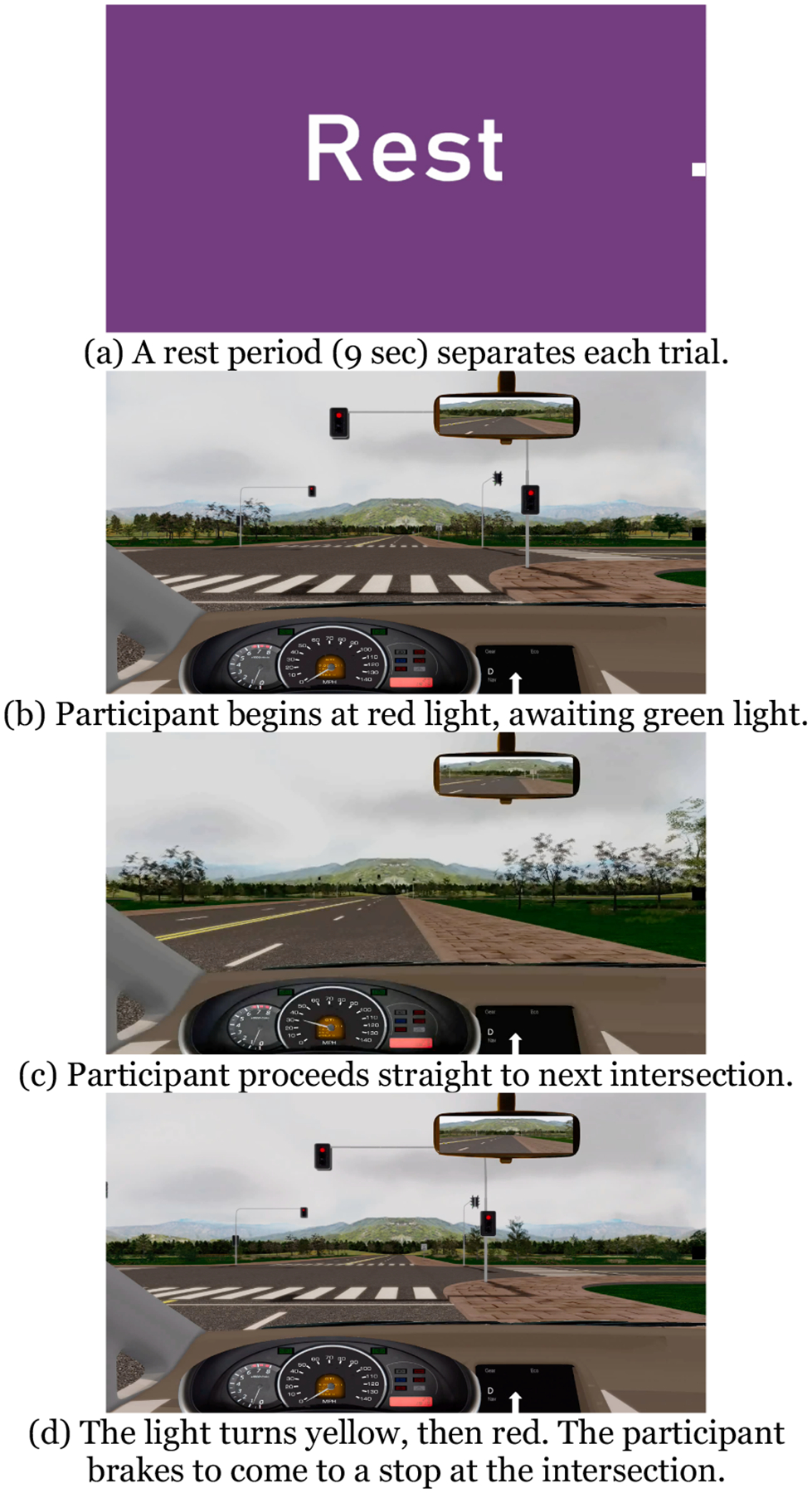
Traffic Light Braking Task.

The Traffic Light Braking task is depicted in Fig. 4. During this low cognitive workload driving task, the participant begins stopped at a red light. Once the light turns green, the participant drives straight to the next intersection, where the traffic turns yellow then red, requiring the participant to brake to a stop. No ambient traffic or distractions are present. The task is repeated for 20 trials, with a 9 sec rest period between each trial. Participants were provided two practice trials and were instructed to drive to the next traffic light while maintaining speed and lane position.

#### 2.5.3. Lead Car Braking task

**Fig. 5.**
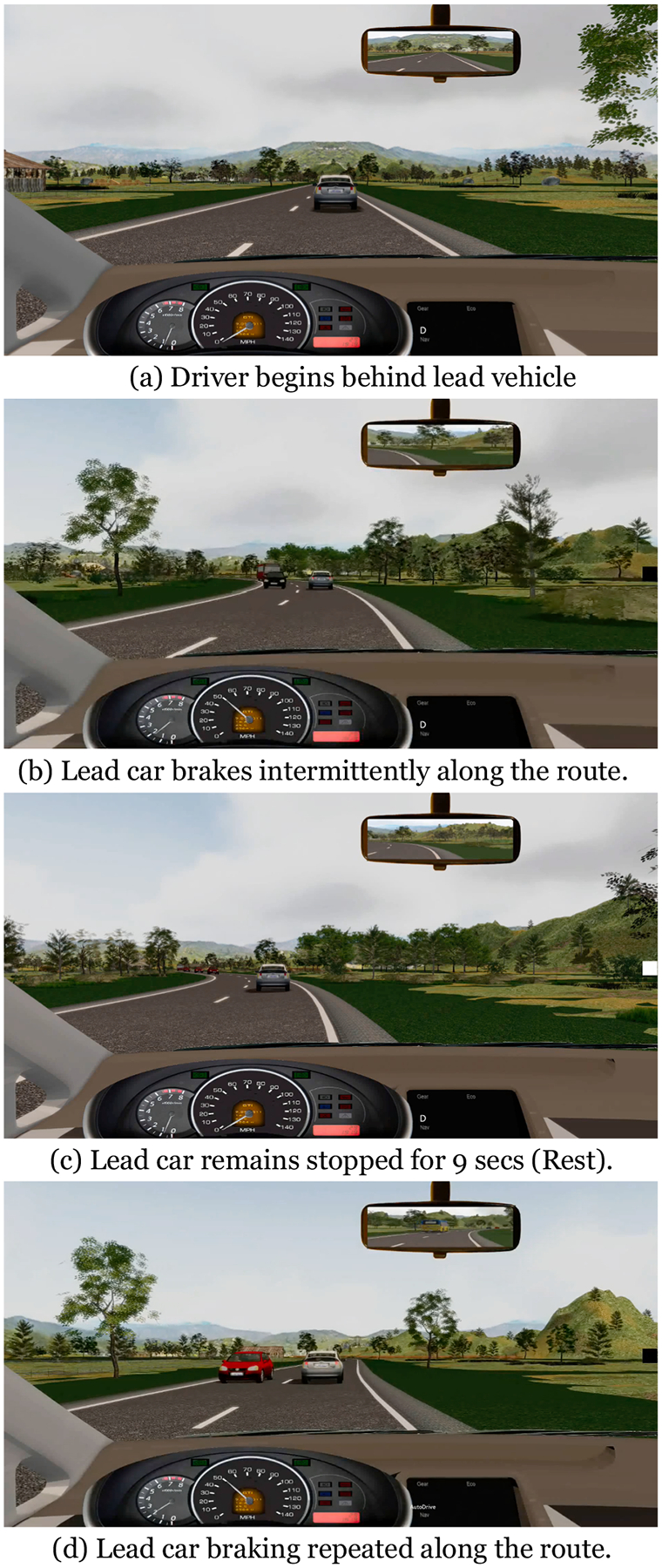
Lead Car Braking Task.

The Lead Car Braking task is depicted in Fig. 5. During this high cognitive workload task, the participant follows a lead car at varying speeds, on a curving highway with oncoming traffic. The participant only controls the brake; steering and throttle are controlled by the simulation to control for exposure to the lead vehicle braking. The participant must be prepared to brake in response to the lead car brake lights at irregular intervals along the route. The lead vehicle brakes 14 times along the route. Each braking event is followed by a “rest” period, where the participant’s and lead vehicles remain stopped for nine seconds, until the lead car begins moving again, and the participant’s vehicle follows.

### 2.6. MEG analysis

The Synthetic Aperture Magnetometry (SAM) beamformer algorithm (Gaetz et al., 2010; Vrba & Robinson, 2001) was used for source localization. SAM produces noise-normalized differential power values (pseudo-t statistics) by contrasting activity in event-related “active” windows with matched baseline periods. Based on prior literature, we examined modulations in beta-band event-related desynchronization (14–30 Hz), motor-related gamma synchronization (60–90 Hz), and frontal midline theta activity (3–9 Hz) in relation to driving task events. Time-frequency responses were visually inspected to define precise analysis windows. Further technical details of preprocessing steps, beamformer implementation, and parameter choices are provided in Walshe et al., (2023).

#### 2.6.1. Defining periods of low and high cognitive control

To identify eye-tracking metrics related to periods of increased cognitive control for the Traffic Light Braking task, we examined (1) a *Coasting* (Lo) period: 4 s of steady-state driving between acceleration and braking when the gas pedal showed minimal variation (i.e. the period requiring minimal cognitive control over behavior while still operating the vehicle) and (2) a *Braking* (Hi) period: a previously established 4 s window beginning at the onset of braking in response to an upcoming red light where there is a distinct increase in FMT activity for cognitive control over driving behavior (Walshe et al., 2023). Based on these previously established periods, similar periods of Lo and Hi cognitive control over behavior were defined for the Unanticipated Steering and Lead Car Braking tasks:

- *Coasting* (Lo) – a 4 s period between acceleration and steering when the gas pedal and steering wheel exhibited the least amount of variation
- *Steering* (Hi) – a 4 s period beginning at the onset of steering in response to the wind gust where a distinct increase in FMT activity for cognitive control over driving behavior was observed
- *Following* (Lo) – a 4 s period prior to lead vehicle braking when the steering wheel exhibited the least amount of variation
- *Braking* (Hi) – a 4 s period beginning at the onset of braking in response to the lead vehicle where a distinct increase in FMT activity for cognitive control over driving behavior was observed

To confirm that the *Steering* and *Braking* periods represented a period of elevated cognitive control relative to *Coasting* and *Following* among these participants, a frequency-specific differential beamformer-based spatial-filter analysis was used to compare FMT activity (3–9 Hz) (Sakihara et al., 2014; Walshe et al., 2023) contrasted against a 4 s baseline window (4–8 sec) during the 9 s rest period prior to each trail. FMT activity was visually assessed across all participants. For more details on this approach, see Walshe et al.(2023).

### 2.7. Eye-Tracking metrics

Fixation count, mean fixation duration, mean horizontal and vertical gaze position, mean saccade amplitude, and horizontal and vertical spread of search were computed during the *Coasting/Following* and *Braking/Steering* periods for all trials, all participants for the three driving tasks. These eye-tracking metrics were chosen based on prior literature linking these metrics to situational awareness and cognitive control during driving (Foy et al., 2016; Foy & Chapman, 2018; Harbluk et al., 2007; Liang et al., 2021; Recarte & Nunes, 2003; Yuen et al., 2021). Furthermore, given the long-term goal of incorporating real-time detection of cognitive control via eye-tracking in-vehicle (where the visual stimuli in driving scene are unknown), eye-tracking metrics were further narrowed to include only “scene-independent” metrics, or eye-tracking metrics that do not rely on defined areas of interest (AOIs) in the visual scene.

### 2.8. Data Reduction

Of the 11 participants, seven completed all three tasks and four completed only the Traffic Light Braking task and Lead Car Braking tasks, resulting in 140 available trials for the Unanticipated Steering task and 220 trials each for the Traffic Light Braking and Lead Car Braking tasks. *Coasting/Following* (*n* = 136) and *Braking/Steering* (*n* = 125) periods with more than 20 % missing eye-tracking data were excluded from the analysis. The final dataset consisted of 115 *Coasting* and 115 *Steering* periods in the Unanticipated Steering task, 187 *Coasting* and 195 *Braking* periods for the Traffic Light Braking task, and 142 *Following* and 145 *Braking* periods in the Lead Car Braking task.

### 2.9. Statistical analysis

The on-screen gaze position and fixation/saccade markers during the *Coasting/Following* and *Braking/Steering* periods were imported into MATLAB 2021b (MathWorks Inc) for analysis. Paired sample t-tests were used to compare sample means (*Coasting/Following* vs. *Braking* or *Steering*) across the eye-tracking metrics. To quantify the magnitude of observed differences between Lo and Hi cognitive control periods, Cohen’s d effect sizes were calculated for each paired comparison using the pooled standard deviation of the two conditions.

## 3. Results

### 3.1. Unanticipated Steering task

Participants successfully returned to the lane for all trials. Increased FMT activity was observed in all participants during the *Steering* period. Mean (±SD) fixation count, fixation duration, mean gaze position, saccade amplitude, and spread of search during *Steering* and *Coasting* are shown in Table 1. Vertical mean gaze was significantly higher, horizontal and vertical spread of search (p < 0.00) were significantly narrower, and saccade amplitude (p < 0.01) was significantly smaller *Steering* than *Coasting*. A trend (p = 0.05) toward longer mean fixation duration during *Steering* was also observed. Vertical mean gaze was significantly higher during *Steering* than *Coasting* (p < 0.01, d = 1.08), and horizontal and vertical spread of search were significantly narrower (p < 0.01, d = 2.18 and d = 2.01, respectively). Saccade amplitude was also significantly smaller during *Steering* (p < 0.01, d = 1.55). A trend toward longer mean fixation duration during Steering was observed (p = 0.05, d = 0.65), and horizontal mean gaze showed a non-significant increase (p = 0.08, d = 0.88).

### 3.2. Traffic Light Braking task

Participants successfully braked at the traffic light for all trials. Increased FMT activity was observed in all participants during the *Braking* period. Mean (±SD) fixation count, fixation duration, mean gaze position, saccade amplitude, and spread of search during *Braking* and *Coasting* are shown in Table 2. Fixation count (p < 0.05, d = 0.56) was significantly lower, vertical spread of search (p < 0.05, d = 0.81) was significantly narrower, and horizontal/vertical mean gaze (p < 0.01, d = 1.45 and 0.99, respectively) was significantly different during *Braking* than *Coasting*. A trend (p = 0.06, d = 0.67) toward longer mean fixation duration during Braking was also observed. Horizontal spread of search (d = 0.27) and saccade amplitude (d = 0.25) showed small effect sizes, suggesting modest changes in scanning behavior.

### 3.3. Lead Car Braking task

Increased FMT activity was observed in all participants during the *Braking* period. Mean (±SD) fixation count, fixation duration, mean gaze position, saccade amplitude, and spread of search during *Braking* and *Following* are shown in Table 3. Fixation count was significantly (p <0.05) lower during *Braking* than *Following*. A trend (p = 0.09) toward longer fixation duration during *Braking* was also observed. Fixation count was significantly (p < 0.05, d = 0.39) lower during *Braking* than *Following*. A trend (p = 0.09, d = 0.44) toward longer fixation duration during *Braking* was also observed. Other metrics, including mean gaze position (horizontal: d = 0.33; vertical: d = 0.09), spread of search (horizontal: d = 0.32; vertical: d = 0.06), and saccade amplitude (d = 0.26), showed small effect sizes, consistent with the similar visual demands of the *Following* and *Braking* periods.

## 4. Discussion

The goal of this proof-of-concept study was to establish the utility of adding eye-tracking to a novel paradigm of combined MEG neuroimaging with simulated driving, paving the way for future research to identify eye-tracking metrics that proxy cognitive control over behavior during driving. Establishing specific eye-tracking metrics that proxy cognitive control during driving will help inform the development of novel in-vehicle technology that target cognitive errors. The findings support our hypothesis that integrating eye-tracking into this paradigm can help establish scanning behavior metrics as a proxy for measures of cognitive control over behavior during driving.

The observed differences in eye-tracking metrics between Lo and Hi periods for each driving task correspond to expected changes in eye-tracking that would accompany cognitive control over behavior. In the Unanticipated Steering task, the narrower spread of search and shorter saccade amplitude correspond to increased focus on the lane markings when steering to return the vehicle to the center of the lane. In the Traffic Light Braking task, the observed decrease in fixation count along with narrow spread of search correspond to increased focus on the traffic signal during braking. Similarly, the higher and more rightward mean gaze represents increased focus on the traffic signals during braking. Finally, the lower fixation count in the Lead Car Braking task corresponds to increased attention on the lead car when braking.

Despite the Lead Car Braking task being the most complex task, only fixation count was significantly lower during braking then steering; a corresponding trend (p = 0.09) toward longer fixation duration was also observed. Given the similarity between the *Following* and *Braking* periods – both *Following* and *Braking* require the participant to remain focused on the lead vehicle – it is not surprising that only minimal differences were observed. The decreased fixation count during *Braking* suggests a modest increase in attention to brake appropriately within the following distance.

To further contextualize the observed differences in eye-tracking metrics between Lo and Hi cognitive control periods, we calculated Cohen’s d effect sizes for each comparison. In the Unanticipated Steering task (Table 1), large effect sizes were observed for vertical mean gaze (d = 1.08), horizontal and vertical spread of search (d = 2.18 and 2.01, respectively), and saccade amplitude (d = 1.55), indicating substantial shifts in scanning behavior during steering. These findings suggest that participants narrowed their scanning behavior and focused more intensely on lane markings when responding to unexpected wind gusts, consistent with increased cognitive control demands. In the Traffic Light Braking task (Table 2), several metrics demonstrated moderate to large effect sizes, including mean gaze position (horizontal: d = 1.45; vertical: d = 0.99), fixation duration (d = 0.67), and vertical spread of search (d = 0.81), suggesting substantial shifts in attention during braking. These findings reinforce the interpretation that braking in response to a traffic signal elicits a focused and deliberate scanning strategy indicative of increased cognitive control. In the Lead Car Braking task (Table 3), effect sizes were generally smaller, with fixation count (d = 0.39) and fixation duration (d = 0.44) showing modest differences. This aligns with the behavioral similarity between the *Following* and *Braking* periods in this task, both of which require sustained attention to the lead vehicle. The smaller effect sizes in this context suggest that while cognitive control is elevated during braking, the visual demands may not differ dramatically from the preceding period. Overall, these effect sizes support the notion that eye-tracking metrics vary meaningfully with cognitive control demands, and their magnitude is task-dependent.

While no single eye-tracking metric consistently differed between the Lo and Hi periods across the different tasks, this variability does not undermine the reliability of eye-tracking as a proxy for cognitive control. Rather, it highlights that scanning behavior is highly context-dependent, shaped by the specific demands of each driving scenario. This task-specific variation suggests that driver monitoring systems should prioritize detecting dynamic changes in eye-tracking metrics relative to driving scenario, rather than relying on fixed thresholds; a lack of change during more complex driving scenarios could indicate cognitive inattention. This aligns with findings from Abuzwidah et al. (2024), who demonstrated that connected and autonomous vehicles perform more safely and efficiently under adverse weather conditions when equipped with adaptive systems. Their work underscores the importance of designing driver monitoring systems that are responsive to environmental context and capable of maintaining reliability across diverse driving scenarios. Moreover, integrating eye-tracking with vehicle telematics, such as steering input, braking force, and lane position, may enhance the robustness of cognitive state detection by providing complementary behavioral indicators. Previous research highlighted the importance of combining multiple data sources to improve predictive accuracy of machine learning models. For example, Ruangkanjanases et al. (2024) applied a multilayer perceptron neural network to traffic accident data, demonstrating that combining multiple features, such as vehicle type, time of day, and accident type, enhanced predictive accuracy. Their findings support the utility of multimodal approaches, suggesting that combining eye-tracking metrics with vehicle telemetry could improve the sensitivity and specificity of systems designed to detect real-time lapses in cognitive attention. Similarly, Abdul Razak et al. (2024) developed a non-invasive ECG-based system to monitor heart rate variability (HRV) and classify driver cardiac health using machine learning. Their findings support the feasibility of physiological sensing for real-time driver monitoring and highlight the potential for integrating biometric signals with eye-tracking and vehicle telemetry to enhance the sensitivity of cognitive state detection.

These findings also address limitations observed in prior neuroimaging and driving paradigms. Studies using fNIRS, fMRI and EEG have largely focused on distraction and multitask paradigms to stress cognitive workload to see neural mechanisms, with limitations in temporal (fNIRS, fMRI) and spatial (EEG) resolution for real-time detection of cognitive control during regular driving (Aygun et al., 2022; Foy et al., 2016; Foy & Chapman, 2018; Liu et al., 2023; Wascher et al., 2023). In contrast, our MEG-based approach offers high temporal and spectral resolution, enabling precise identification of cognitive control states during discrete driving events. By integrating eye-tracking, this study advances the field by linking behavioral metrics to validated neural markers of cognitive control in real time.

### 4.1. Strengths and limitations

A key strength of this study is the integration of MEG neuroimaging with eye-tracking in a controlled, event-based driving paradigm. This allowed for precise temporal alignment of neurocognitive and behavioral data. However, several limitations warrant discussion. First, the prescribed seating position and lack of head motion permitted in the MEG may have influenced eye-tracking. While the upright seated posture used in the MEG is more naturalistic than the supine position used in prior fMRI studies, future work should explore whether differences in eye-tracking between Lo and Hi periods are observed in more naturalistic seating postures that allow additional head motion. That said, we do not believe that the restricted head motion in the MEG environment adversely affected the participant’s eye-tracking metrics. Given that lateral or panoramic projection is not technically feasible in the MEG, the driving scene was presented on a single front-panel, back-projected screen, allowing participants to maintain their view of the driving scene using eye movements alone. Consequently, the lack of head motion likely had minimal impact our results, as our eye-tracking metrics (e.g., fixation duration, mean gaze location) reflect the result of visual attention regardless of whether it was achieved through eye movements alone or in combination with head motion. In fact, the restriction of head movement may be considered a methodological strength in this proof-of-concept study, as it eliminates a potential confound and allows for more precise quantification of the relationship between eye-tracking metrics and cognitive control processes measured by MEG. Nonetheless, we acknowledge that ecological validity would benefit from future studies incorporating more naturalistic head and eye movement strategies in simulator or on-road environments.

Additionally, two of our driving tasks represent relatively simplistic driving scenarios: the Traffic Light Braking and Unanticipated Steering tasks. These simplistic tasks serve as a useful probe of increased cognitive control over behavior during driving, without the influence of additional cognitive demands (e.g. ambient traffic). While our Lead Car Following task included ambient traffic, no scenario required the driver to perform more complex driving tasks, such as navigating an intersection, anticipating hidden hazards, or dealing with adverse weather conditions. Future work should explore the relationship between eye-tracking and cognitive control over behavior in more complex driving tasks.

The order of the driving tasks was not randomized across participants. This was done deliberately to provide increasing complexity in terms of vehicle control and attentional demands: vehicle control only with no specific AOI in the Unanticipated Steering task, vehicle control with a static AOI in the Traffic Light Braking task, and vehicle control with a dynamic AOI in the Lead Car Braking task. While this approach has the advantage of allowing the participant to gradually ramp up the task demand, it did not allow us to control for the influence of implicit learning across the driving tasks. Additionally, presenting tasks in a fixed order may introduce confounding effects related to learning, adaptation, or fatigue, which could influence eye-tracking or cognitive control responses. Future work should explore varying the order of these three tasks in a larger sample.

Another limitation of this proof-of-concept study is that we did not systematically test alternative sets of eye-tracking metrics or perform sensitivity analyses on our definitions for Lo and Hi periods of cognitive control. The eye-tracking metrics used were selected based on prior literature linking them to situational awareness and cognitive control during driving. Given our long-term goal of enabling real-time detection of cognitive control via eye-tracking in naturalistic driving environments, we further narrowed our focus to “scene-independent” metrics, or those that do not rely on predefined AOIs in the visual scene. This design choice reflects the practical constraints of in-vehicle driver monitoring systems, where AOIs may be unknown or highly variable. While this approach enhances translational relevance, it may exclude other potentially informative metrics. Future work should explore a broader range of eye-tracking metrics and conduct sensitivity analyses on the selected window for identifying Lo and Hi periods of cognitive control to determine the influences on detection of cognitive control states.

As this study was exploratory in nature and aimed to establish the feasibility of integrating eye-tracking with MEG during simulated driving, we did not apply post-hoc corrections for multiple comparisons. Our primary objective was to identify potential eye-tracking metrics that may serve as proxies for cognitive control, rather than to confirm specific hypotheses with strict inferential thresholds. Accordingly, we reported p-values in the 0.05–0.09 range as “trends” to transparently convey potentially meaningful patterns that merit further investigation. To aid interpretation, we have included effect sizes (Cohen’s d) for all comparisons, which provide additional context regarding the magnitude of observed differences and support the relevance of these findings despite the absence of statistical significance after correction.

Finally, participants were younger than typical driving age in this initial proof-of-concept study. Younger adolescents were chosen to limit the influence of driving experience on FMT activity. Although this group is not legally permitted to drive, their inclusion allowed us to isolate core neurocognitive processes related to cognitive control without the confounding effects of learned driving behaviors. The younger age of our participants, combined with the small sample size, may limit the generalizability of these findings. However, these results confirm the utility of this paradigm, which will be used in future work to explore eye-tracking metrics related to periods of cognitive control during driving across varying age and driving experience. Future work will specifically target novice licensed drivers (ages 16–20), who represent a primary at-risk group for driving errors, to assess the applicability of this paradigm in higher-risk populations. We anticipate that expanding the sample size will provide more robust evidence of eye-tracking metrics that vary between periods of high and low cognitive control. Furthermore, including older adolescent and licensed drivers will reveal developmental and experience-based differences in both eye-tracking metrics and neural markers of cognitive control. For example, more experienced drivers may demonstrate more efficient scanning behavior and reduced FMT activity during similar tasks, reflecting maturation of executive function and driving skill.

### 4.2. Future directions

While the current study establishes the feasibility of integrating eye-tracking with MEG to characterize scanning behavior during periods of elevated cognitive control during simulated driving, several important directions remain for future research. First, replicating this protocol with a larger sample and expanding the paradigm to include older adolescents and licensed young adult drivers will allow us to examine how eye-tracking and cognitive control evolve with age and driving experience. We anticipate that more experienced drivers may show more efficient scanning strategies and potentially reduced FMT activity during similar tasks, reflecting maturation of executive function and driving skill.

Second, future work should explore the use of machine learning approaches to classify Hi vs. Lo cognitive control periods based on multivariate eye-tracking features. As a preliminary step, our current dataset includes multiple eye-tracking metrics (e.g., fixation count, duration, gaze position, saccade amplitude, spread of search) that could serve as input features for supervised classification models. These features could be extracted and labeled based on MEG-defined cognitive control periods, enabling training of classifiers such as support vector machines, random forests, or neural networks. We anticipate that such models could achieve meaningful accuracy in distinguishing periods of elevated or lapsed cognitive control over behavior, paving the way for real-time detection of cognitive lapses using driver monitoring systems.

Third, integrating additional physiological measures, such as heart rate variability, could provide complementary indices of cognitive workload and autonomic regulation. Combining these signals with MEG and eye-tracking may yield a more robust understanding of the neurophysiological basis of driving behavior.

Fourth, future studies should incorporate more ecologically valid driving scenarios that reflect the complexity of real-world driving. While the current tasks were intentionally simplified to isolate cognitive control over behavior, actual driving involves distractions, intersections, dynamic traffic, and hidden hazards. Expanding the paradigm to include these elements will allow for a more comprehensive assessment of eye-tracking and cognitive control under realistic conditions. However, doing so presents several challenges, including increased variability in driver responses, difficulty in precisely time-locking neural and behavioral events, and the need for more sophisticated simulation environments that remain compatible with neuroimaging constraints. Despite these challenges, we anticipate that such extensions will enhance the translational relevance of this work and support the development of driver monitoring systems capable of detecting cognitive lapses.

Finally, once feasibility is firmly established, future studies should test this paradigm in more naturalistic driving environments. This will enhance ecological validity and assess how well laboratory-derived metrics generalize to real-world driving contexts. A key challenge in translating this work to real-world applications is achieving reliable, real-time detection of cognitive control states in-vehicle using eye-tracking. Recent advances in pupil estimation, such as the semantic segmentation approach developed by Kurdthongmee & Kurdthongmee (2024), offer promising solutions by enabling fast and accurate pupil localization even under variable lighting and camera conditions. Together, these extensions will strengthen the translational potential of this work and support the development of in-vehicle driver monitoring systems capable of detecting and responding to cognitive deficits in real time.

## 5. Conclusions

Our findings suggest that eye-tracking metrics may be a useful proxy of cognitive control during driving. These findings support a path toward in-vehicle driver monitoring systems that infer cognitive deficits in real time by linking frontal-lobe indices to eye-tracking–derived driver behaviors. Future research should expand the sample to include licensed drivers, incorporate more complex and ecologically valid driving scenarios, and explore machine learning approaches for real-time classification of cognitive control states. Additionally, integrating eye-tracking with vehicle telemetry and physiological signals may enhance the robustness and reliability of in-vehicle safety systems.

## Figures and Tables

**Table 1:** Comparison of Eye-Tracking Metrics during Coasting and Steering for the Unanticipated Steering Task.

Metric		Unit	Coasting	Steering	p-value	Cohen’s d
Fixation Count		#	12.7 ± 5.6	11.6 ± 8.4	0.40	0.15
Fixation Duration		ms	314 ± 100	425 ± 200	0.05	0.65
Mean Gaze	Horizontal	pxls	843 ± 97	926 ± 92	0.08	0.88
	Vertical	pxls	789 ± 67	697 ± 97	0.00*	1.08
Spread of Search	Horizontal	pxls	165 ± 28	107 ± 25	0.00*	2.18
	Vertical	pxls	176 ± 36	111 ± 28	0.00*	2.01
Saccade Amplitude		pxls	169 ± 39	111 ± 37	0.01*	1.55

**Table 2:** Comparison of Eye-Tracking Metrics during Coasting and Braking for the Traffic Light Braking Task.

Metric		Unit	Coasting	Braking	p-value	Cohen’s d
Fixation Count		#	11.7 ± 3.4	9.7 ± 3.7	0.02*	0.56
Fixation Duration		ms	339 ± 125	429 ± 146	0.06	0.67
Mean Gaze	Horizontal	pxls	839 ± 55	932 ± 72	0.00*	1.45
	Vertical	pxls	738 ± 68	652 ± 100	0.00*	0.99
Spread of Search	Horizontal	pxls	160 ± 33	147 ± 59	0.46	0.27
	Vertical	pxls	174 ± 24	147 ± 38	0.04*	0.81
Saccade Amplitude		pxls	176 ± 34	162 ± 64	0.46	0.25

**Table 3:** Comparison of Eye-Tracking Metrics during Coasting and Braking for the Lead Car Braking Task.

Metric		Unit	Coasting	Following	p-value	Cohen’s d
Fixation Count		#	10.6 ± 3.9	9.2 ± 2.9	0.04*	0.39
Fixation Duration		ms	379 ± 105	441 ± 162	0.09	0.44
Mean Gaze	Horizontal	pxls	943 ± 54	927 ± 38	0.34	0.33
	Vertical	pxls	589 ± 77	582 ± 76	0.36	0.09
Spread of Search	Horizontal	pxls	83 ± 30	73 ± 33	0.14	0.32
	Vertical	pxls	50 ± 23	52 ± 46	0.29	0.06
Saccade Amplitude		pxls	92 ± 38	81 ± 45	0.32	0.26

## Data Availability

Data will be made available on request.
